# Translocation as a Novel Approach to Study Effects of a New Breeding Habitat on Reproductive Output in Wild Birds

**DOI:** 10.1371/journal.pone.0018143

**Published:** 2011-03-30

**Authors:** Claudia Burger, Christiaan Both

**Affiliations:** Animal Ecology Group, Centre for Life Sciences, University of Groningen, Groningen, The Netherlands; University of Jyväskylä, Finland

## Abstract

Environmental conditions under which species reproduce have major consequences on breeding success and subsequent fitness. Therefore breeding habitat choice is ultimately important. Studies rarely address the potential fitness pay-offs of alternative natural breeding habitats by experimental translocation. Here we present a new tool to study fitness consequences of free living birds in different habitats. We translocated a migratory passerine, the pied flycatcher (*Ficedula hypoleuca*), to a novel site, where pairs were subjected to a short stay (2–4 days) in a nest box-equipped aviary before being released. We show that it is technically possible to retain birds in the new area for breeding, allowing the study of reproductive consequences of dispersal under natural conditions. The translocation resulted in an extension of the interval between arrival and egg laying of four days, highlighting the importance of having an adequate control group. Clutch size and nestling parameters did not differ significantly between translocated and unmanipulated females, which suggests that the procedure did not affect birds in their reproductive performance later on. This method could be applied broadly in evolutionary and ecological research, e.g., to study the potential fitness benefits and costs for dispersing to more northern latitudes as a way of adapting to climate change.

## Introduction

Environmental conditions have a great effect on avian breeding success and birds should be adapted to choose a location that maximizes fitness. Therefore, habitat choice of a bird is considered as a major life-history decision [1], [2]. The fitness consequences of habitat choice depend on physical and ecological features of the habitat, how well birds are adapted to these local circumstances, and the competition they experience. The idea that individuals are locally adapted to their breeding habitat assumes that they perform less in fitness terms if they are forced to breed at another place. This assumption is rarely tested under natural conditions outside isolated islands [3], because it appears difficult to force individuals to breed at a different place, and study their fitness compared to unmanipulated controls. Here we present a novel experimental procedure to study fitness consequences of avian habitat choice under natural conditions.

Habitat choice of a bird should have evolved to maximise fitness pay-offs [4] and can be based on, for example, innate preferences [5], previous experience or public information [6]. However, a birds' habitat choice might become maladaptive if adopted cues do not allow to track changes in the environment. In seasonally changing environments, the precise timing of arrival and breeding relative to the timing of other organisms in that environment is often crucial [7]. In addition, current climate change differentially shifts the timing of the annual cycle of many organisms leading to mismatches between cycles of prey and predator [8], [9]. This mismatch is particularly acute in long-distance migrants breeding in temperate forests: their arrival and breeding dates advanced less than the peak abundance of caterpillars [10], [11]. Consequently, they fail to profit from the short food peak in spring, with possible fitness consequences and population declines [12], [13].

If birds are unable to adjust sufficiently to changes in one habitat, dispersal to a different habitat might be an advantageous mechanism. Our aim was to develop a method to experimentally study the consequences of this potential mechanism. Successful adaptation to climate change is possible without a change in timing by dispersing to habitats that show less seasonality in food abundance or a later food peak. To overcome the problem of arriving and breeding too late under conditions of severe climate warming, individual birds could also move to more northerly breeding areas where spring starts later. This could be advantageous for the individual by increasing its reproductive success. In addition, genes for earlier migration introduced by birds normally breeding at more southerly latitudes could also facilitate adaptation in the northern population [14] if they result in a better match with the phenology of their main prey. Although those genes may be beneficial in a northern breeding area, there are potentially high costs involved in moving north. Evolutionary costs can be the break-up of co-adapted gene complexes and outbreeding, which can result in a loss of beneficial local adaptations [15]–[17]. Introduced individuals lack experience with the local habitat and its associated food sources and predators [18], [19]. In order to find out how important northward dispersal could be as an adaptation to climate change, we need to study fitness correlates of dispersing individuals.

Studies comparing the performance of philopatric birds with that of long-distance dispersers hardly exist, mainly because of the difficulties to track birds [20]. One example is a study by Hansson *et al.* [21] where stable isotope analysis was used to identify immigrating long-distance dispersers. Lifetime reproductive success was found to be lower in long-distance dispersers, but the authors could not exclude non-random dispersal (e.g. greater movements of low-quality individuals) as a potential explanation of this result [22].

It is still unclear how important long-distance dispersal is in our focal species, the pied flycatcher (*Ficedula hypoleuca*), but it has been suggested to be more common than usually thought [23]. Current data show that the migratory pied flycatcher does occasionally perform extensive natal dispersal [24], [25]. However, reports of natal dispersal over hundreds of kilometers are rare (but see [26]). Moreover, the low detection rate of such movements inhibits the study of fitness consequences of long-distance dispersal in this species under natural conditions.

Artificial translocation, and release of birds into a new breeding area, can be used to mimic long-distance dispersal. This is a common tool in conservation biology for the reintroduction of species [27], [28]. However, the immediate release of pied flycatchers at a distant site (250 km away) resulted in a high proportion of birds disappearing from this new location (>80%, [23]), therefore making it difficult to compare breeding performance of translocated and philopatric birds. In another study, pied flycatchers were translocated into a different habitat by gradually moving their nest box during nest building [29]. Only short distances (around 50 m in this case) can be covered with this technique though.

In this paper we present a new set-up which allows for the study of reproductive consequences of long-distance dispersal in free-ranging birds. In the presented pilot experiment, we captured pairs shortly after arrival at the breeding grounds and kept them as pair in a nest box-equipped aviary at a novel site for several days before releasing them again. We performed this pilot experiment during the breeding season of 2009 with the aim to evaluate the set-up for future experiments on long-distance dispersal. We tried to: (a) confirm, that translocated birds stay at the new location and breed there, and (b) estimate the impact of the set-up on the reproductive behaviour of the birds.

## Results

Non-systematic observations of captive birds revealed that most males soon started advertising the new box to their partner and most females were seen nest building before release. Of the nine translocated females, six started breeding in the new box they were assigned to, while three other females moved short distances (95, 110 and 155 m) to a different box to breed with a novel male. Of the eight males, four were recaptured at the nest of their aviary partner. One male moved (145 m) within the plot to another box and a second male returned to the place of capture (13 km) and bred there. The two remaining males (of which one was used twice) disappeared and were not recaptured. One of their females bred in the box of release, and the other moved to another box.

**Figure 1:**
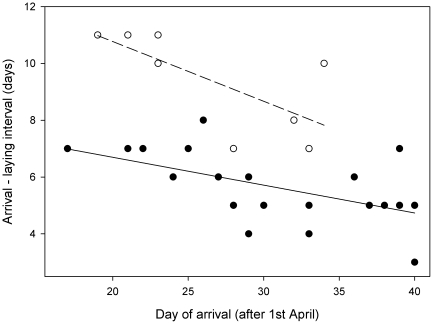
Effect of translocation on the interval between female arrival and laying date in pied flycatchers. Interval between arrival date of females in the original breeding area and first egg date was used. Translocated nests: open circles and dashed regression lines. Control nests: filled circles and solid regression lines.

**Figure 2:**
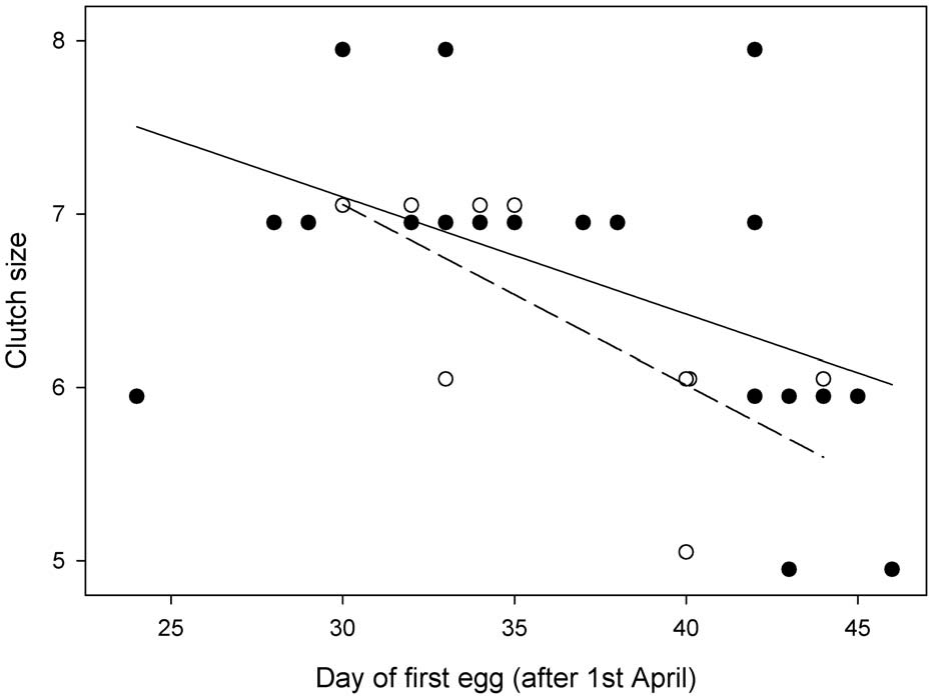
Effect of translocation on the correlation between laying date and clutch size in pied flycatchers. First egg date was used as laying date. For graphical reasons only, original data points were shifted 0.05 lower (control) or 0.05 higher (translocated) along the y-axis. Regression lines were calculated from original data.

All three artificially formed pairs broke up after release, while four of the six original pairs stayed together. The likelihood to move away from the release site also was higher for birds that bred in the area of capture in the previous year (males and females, only one out of six stayed at the assigned box), while birds not breeding in the area before stayed more often at their assigned box (nine out of eleven birds). The time interval between female arrival at the original site and laying of the first egg at the new site significantly declined with date (F_1,29_ = 11.78, p<0.01) and was on average 9.7 (±1.3 S.D.) days (Fig. 1). The interval was significantly longer compared with unmanipulated females which commenced laying on average 5.6 (±1.2 S.D.) days after arrival (F_1,30_ = 73.04, p<0.001). Clutch size declined significantly with date (F_1,31_ = 17.77, p<0.001), but did not differ between translocated and control nests (F_1,30_ = 1.62, p = 0.21; translocated: n = 9, mean ± SD: 6.33±0.71, control: n = 24, mean ± SD: 6.63±0.88, Fig. 2). Mean fledging weight at age 12 days (translocated: 13.8±1.4 g, control: 14.6±1.0 g; mean±S.D.): and number of fledglings (translocated: 5.0±1.8, control: 5.5±1.8; mean ± S.D.) did not show large differences, but because of another experiment taking place after laying in the control group, sample sizes were very limited (six nests) and we therefore do not give the statistics here.

In 2010, we recaptured four of the translocated males (50%) and one female (11%), all in their original area. Three of these males had successfully bred in the release area, as had the female. Interestingly, two of these males did not breed in the original site before the experiment, but did move back after successful breeding in the new site.

## Discussion

The translocation of pairs of pied flycatchers to a site 13 km of their original site, resulted in all nine translocated females to start breeding within the release plot, confirming that our translocation procedure is suitable for measuring effects of dispersal on reproductive output. Previous breeding experience seemed to affect a birds' decision to stay at the exact place of release. It is known that adult male pied flycatchers show relatively high site-fidelity to a previous breeding location [30], and therefore males with local breeding experience may return more often to their former breeding site. Using inexperienced first-year breeders will thus likely increase translocation success. Males seemed to be less prone to stay than females, but males could have been missed even if they stayed in the area and when their females moved away to breed in another nest box. Furthermore, males could have been affected more by interference with neighbouring males due to the short distances between boxes (<50 m in some cases). Familiarity with the partner seemed to increase the probability of the pair remaining together, which was not found for other species that were translocated [31]. The success of these experiments thus likely depends on the selection of individuals depending on their previous history.

The interval between arrival at the original site and egg laying was about four days longer compared to the control group. This was most likely caused by the translocation or the aviary phase. Therefore, when applying this set-up, it is important to use a control group of birds receiving the same treatment whilst being translocated randomly, for example within the original breeding area. In addition, our result also indicates that females did not use the 2–4 days of supplemental feeding to advance laying, which has been sometimes found in other food-supplementation experiments of longer duration [32]. Rather, egg laying started several days later than in control birds. The treatment caused no significant difference in clutch size between the groups, when controlling for date, suggesting that the performance of pairs after release was not severely affected by previous procedures but by the local environment experienced.

With our set-up we could solve two main methodological problems of studying consequences of dispersal, randomization and detectability of free-living individuals. The advantage of our approach is that birds breed in a natural environment, the choice of which is at the mercy of the experimenter. This makes observed differences in performance more meaningful and easier to interpret compared to experiments in captivity where *ad libitum* food and exclusion of predators might conceal many effects [33].

Our set-up could have many applications, not only for the study of long-distance dispersal. Correlative evidence suggests that under conditions of climate change it becomes increasingly doubtful if the habitat choice of species is still adaptive [34]. If preferences of species remain stable but environmental conditions change, the preferred habitats might even become ecological traps with serious consequences for populations and species. So far, experimental evidence on the existence of ecological traps is still scarce, especially for birds [35], [36].

Recently, translocations have been suggested as a conservation tool, especially to speed up local adaptation under conditions of climate change [37]. Our study shows that it is technically possible to improve the success of such translocations, although those generally also imply potentially serious, unforeseen risks for an ecosystem that require deliberate consideration. Other applications could be the investigation of consequences of mate choice or experiments manipulating the density of breeding pairs in a plot.

There are, however, species-specific limitations to the suitability of our approach. The aviary set-up is applied easily for small-sized species only, e.g. small passerine birds. Capture of pairs before breeding must be feasible as well. Individuals then need to become attached to the new spot, which is probably achieved best for cavity-nesting species, where birds experience the provided nest box as an indicator of a high-quality territory that should be defended [38]. To avoid competition with other territorial breeding birds, nearby breeding opportunities should be removed or blocked. Pied flycatchers are ideal in this respect, because they only defend a small area around the nest box. Sensitivity of birds to the translocation procedure has to be rather low, as handling effects might otherwise overrule experimental effects on breeding behaviour.

Overall, we conclude that this approach could become an important tool to the study of dispersal and more general for investigating effects of breeding environment on behaviour and reproductive success. A wide array of studies in (behavioural) ecology and evolution would benefit from the ability to remove the linkage between the birds' choice of breeding location and a trait under study, and the ability to force some desired breeding environment for individuals, all while allowing free-ranging, natural breeding.

## Materials and Methods

### Ethical Statement

All work was conducted according to the Netherlands Code of Conduct for Scientific Practice, and under license of the Animal Experimental Committee of the University of Groningen (license DEC-5588A).

### Study Species and Experimental Procedure

Pied flycatchers are long-distance migratory passerines breeding in natural cavities and nest boxes in temperate forests across Europe. They are single-brooded and both parents participate in nestling care [30]. We caught 17 individuals (six pairs, plus three females and two males without their original partner) at our study site in Drenthe, the northern Netherlands, during the early nest-building stage in April and May 2009. Birds were then transported to another nest box area 13 km away. This area offered 100 nest boxes distributed over 0.3 km^2^. Six original pairs and three newly formed pairs (one male was used twice with two different females, after his first partner moved to another male) were released into outdoor aviaries built around a tree with a nest box. Dimensions of the aviaries were 2×2×2 m. Food (mealworms) and water were provided ad libitum on a feeding table, as well as nesting material and perches. The pairs were kept in the aviaries between two and four days and were subsequently released. To keep disturbance at a minimum we removed the netting on all sides of the aviaries, but left the structure in situ until the next day when it was removed. Nests were then monitored throughout the breeding season to collect data on laying date, clutch size, nestling weight and fledging success. The control group consisted of 23 nests, located at the site of release of the experimental birds, that remained unmanipulated until egg laying started. In 17 of these control nests eggs were collected daily after laying and replaced by dummy eggs for another experiment.

### Statistical Analysis

Statistical analysis was done using GLM in R (version R 2.10.0) with normal error structure. Models were ANCOVAs with date as covariate and treatment as factor.
